# User satisfaction with cochlear implant audio processors: population norms derived from a large-scale survey campaign

**DOI:** 10.3389/fpsyg.2026.1817444

**Published:** 2026-09-07

**Authors:** Mareike Billinger-Finke, Dorothée B. Hoppe, Karin A. Koinig, Elena Grandelis, Patrick F. Connolly, Ilona Anderson

**Affiliations:** MED-EL Elektromedizinische Geräte GmbH, Innsbruck, Austria

**Keywords:** APSQ population norm values, audio processor, cochlear implant, device usability, user satisfaction

## Abstract

**Introduction:**

Cochlear implants (CIs) require an external audio processor (AP), which can impose responsibilities and challenges for the user. Monitoring user satisfaction is crucial to identify and address issues that may lead to reduced device usage. The audio processor satisfaction questionnaire (APSQ) was developed to measure user satisfaction across three subscales: user comfort, social life, and device usability.

**Methods:**

We analyzed 861 APSQ responses collected between 2019 and 2023 from CI users in Germany and the UK. Multivariable quantile and ordinal logistic regression models were used to examine factors associated with satisfaction and daily usage duration. Population norm values were established for the total score and subscales.

**Results:**

Median total APSQ scores were high (8.7/10). Males and users with single-sided deafness (SSD) reported slightly lower satisfaction. Off-the-ear (OTE) processors produced marginally higher usability ratings. Higher APSQ scores were strongly associated with longer daily CI usage. Each additional point on the total score was associated with 44% higher odds of using the CI at least 3 h longer per day. SSD and bimodal users showed higher odds of shorter use relative to bilateral CI users. Item-level comparisons indicated greater wearing comfort with glasses for OTE users and with headwear for behind-the-ear (BTE) users.

**Conclusion:**

CI user satisfaction, as measured by the APSQ, is generally high and is positively associated with daily usage duration. The APSQ is a valuable tool for monitoring user experience and identifying populations at risk of reduced CI use. The population norm values facilitate the interpretation of clinically acquired APSQ scores.

## 1 Introduction

Current cochlear implant (CI) designs require the wearing of an external audio processor (AP). These devices necessarily impose a set of responsibilities and burdens on the user. The AP must be protected from damage or loss. It must be kept clean and charged. Wearing it can induce discomfort in some users (Holder et al., 2022). It also acts as a visible marker of disability (Chang and Tucker, 2022) and may not be compatible with one's aesthetics or self-image. These new responsibilities and burdens are usually encountered during a period of intensive auditory rehabilitation; already a challenging and stressful experience.

In extreme cases, the difficulties associated with wearing an AP may lead to total discontinuation of device use. Reported rates of discontinuation vary, but roughly 3%−4% of users completely cease using their device (Calvino et al., 2023), and numbers as high as 11% for pediatric users have been reported (Spencer et al., 2004). Discontinuation represents a lost opportunity for hearing rehabilitation, with attendant social (Shukla et al., 2020; Podury et al., 2023), economic (Huddle et al., 2017; McDaid et al., 2021), and cognitive impacts (Kral and Eggermont, 2007; Loughrey et al., 2018).

For these reasons, it is imperative to monitor the degree to which the user is satisfied with their device. This can allow the early detection of issues which, if left unchecked, may progress to reduced daily usage duration or discontinuation. User satisfaction is an inherently subjective phenomenon. As such, a psychometric instrument is called for.

MED-EL has developed and validated such an instrument: the audio processor satisfaction questionnaire (APSQ) (Billinger-Finke et al., 2020; MED-EL, 2025). This instrument measures device satisfaction across three subscales; *social life, usability*, and *wearing comfort*. It has 15 items and can be completed in 5–10 min. In the initial validation study, it demonstrated good internal consistency (Cronbach's α = 0.84) and test-retest reproducibility (*r* = 0.82), as well as good construct validity, with items loading onto separate factors. It was initially developed in the German language but has since been validated for use in English (Felder et al., 2022) and Arabic (Alasmi et al., 2024).

Since 2019, the APSQ has been distributed with each AP shipped by MED-EL in Germany and the United Kingdom. Users are requested, but not required, to complete and return it to the manufacturer no earlier than 28 days after device activation. Over a four-year period, we have obtained a large corpus of APSQ responses completed by hundreds of users.

The goals of this study were (1) to examine associations of demographic and device characteristics with user satisfaction levels, (2) to establish population norm values for the total score and the subscale scores, (3) to examine associations of user satisfaction levels, as well as demographic and device characteristics, with the reported duration of daily usage, and (4) to compare satisfaction levels between behind-the-ear (BTE) and off-the-ear (OTE) APs for six items we hypothesized might differ between these two AP types.

## 2 Materials and methods

### 2.1 Study ethics

This non-interventional data collection was intended to acquire real-world data about CE-marked medical devices used within the scope of their intended purpose. It did not impose any additional invasive or burdensome procedures. Collection of this data is required for our post-market clinical follow-up activities as mandated by Regulation (EU) 2017/745 (EU MDR). All data were anonymized at source and respondent identity cannot be recovered from the data provided. Questionnaires were returned by CI users on voluntary basis.

### 2.2 The APSQ

The APSQ is a validated patient-reported outcome measure intended to measure subjective user satisfaction with APs (Billinger-Finke et al., 2020). It contains 15 items grouped into three subscales: *social life, usability*, and *wearing comfort*.

Items are in the form of statements to which the user can express their degree of agreement on a visual analog scale. The scale ranges from 0 to 10, where higher ratings indicate greater satisfaction. As visual anchors, the rating zero is labeled *I do not agree at all* and 10 is labeled *I fully agree*. Users can also check a box indicating that a given statement does not apply in their case (for example the item related to wearing comfort with glasses). These data were treated as missing values in the present analysis. Left and right ears are rated separately. The three subscale scores are obtained by averaging the scores over their respective items. The total score is obtained by averaging over all items.

In addition to the rated items, users are also asked to state their hearing status for each ear (*normal hearing* or *hearing loss*), which hearing device is used (*no device, a hearing aid*, or *a hearing implant system*—with separate options for *CI, CI with integrated hearing aid, middle ear implant, bone conduction implant*, or *auditory brainstem implant*). Users are also asked when they received their hearing implant (month/year) and what model of AP they currently use, as well as how many h per day they use their AP. This question had categorical responses: *never, 0–3, 3–6, 6–9, 9–12*, or >*12 h*. Users also provide the current date, their current age, and their gender.

The APSQ can be completed on paper and returned to the manufacturer via post mail, or it can be completed online via a URL provided. The questionnaire is to be completed no earlier than 28 days after AP activation.

### 2.3 Data collection and participants

Data collection started in December 2019. This survey is continuous and ongoing. For the purpose of this analysis, we included all data collected until December 2023. We obtained 1,684 responses during this period, covering 1,296 CI users.

**Figure 1:**
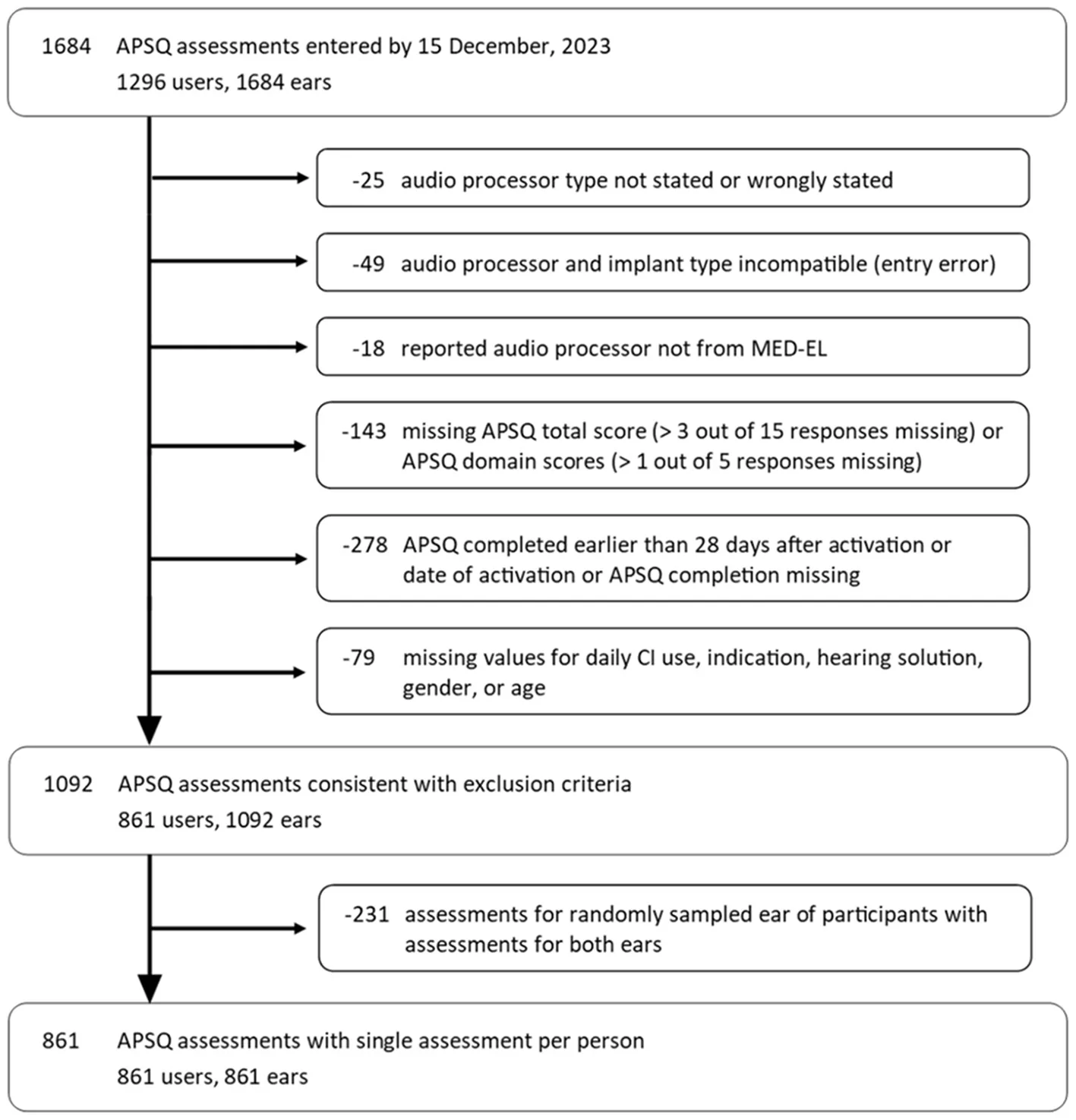
STROBE diagram of APSQ response processing.

Several exclusion criteria were applied (Figure 1). Most of these were used to ensure that there were no missing data or erroneous responses in the outcomes and variables investigated. We excluded responses where the AP was not stated or erroneously stated (e.g., an implant model was stated instead of an AP model). We excluded erroneous responses where the AP and the implant type were not compatible (for example a bone conduction AP paired with a CI). We also excluded responses where the AP was not one manufactured by MED-EL. Responses that were completed prior to 28 days after activation were excluded, as were those where the date of activation or date of APSQ completion were not stated—this was to ensure that only scores of participants with some experience with their AP were included. The APSQ total score was not calculated if more than three of the 15 items were unanswered and subscale scores were not calculated if more than one of the five items were unanswered. We excluded responses with missing information on daily usage duration, AP or implant type, gender, or age. To ensure independence of assessments in cases of bilateral CI users, we selected either the left or right device of bilateral users through random sampling. After these exclusions, 861 responses from 861 CI users remained for analysis.

### 2.4 Outcomes and analysis variables

All analyses were conducted in R version 4.5.1. AP satisfaction was assessed with the APSQ total score and subscale scores, all on a continuous scale from 0 to 10. Daily usage duration was assessed with the self-reported response categories. Given the very small number of responses to “*never*,” we combined the categories *never* and *0–3 h*, yielding five category levels for analysis: *0–3, 3–6, 6–9, 9–12*, and >*12 h*.

We organized user-reported demographic and device characteristics into categories. Audio processor type was categorized as behind-the-ear (BTE) or off-the-ear (OTE) based on reported AP models. Four hearing configurations were defined based on reported hearing status and hearing devices: bilateral hearing loss with two CIs (bilateral CI), bilateral hearing loss with one CI (unilateral CI), unilateral hearing loss with one CI (SSD) and use of a CI with an integrated hearing aid (bimodal). For statistical modeling, age in years was mean-centered and scaled by a factor of 10, so that one unit corresponded to a 10-year difference. This transformation was applied to improve the interpretability of model coefficients. In addition, the few answers (*n* = 11) reporting gender as *other* were not used for the development of statistical models, given that the small category size could not ensure unbiased estimates.

### 2.5 Factors associated with APSQ scores

Multivariable quantile regression models were used to assess the association of demographic and device factors with median APSQ scores. Quantile regression was chosen because it does not require distributional assumptions and was appropriate given the strong negative skew of the APSQ scores. Four separate models were fit; one to model the total score and one to model each of the subscale scores. Each model assessed four fixed main effects: AP type, hearing configuration, gender, and age. No interaction effects were specified. We assessed multicollinearity with generalized variance inflation factor (VIF), which were all below a value of 1.10. Model fitting was performed with the R package quantreg (Koenker, 2025). Standard errors of the model coefficients were bootstrapped with the *xy*-pair method, which were then used to calculate *p*-values.

To check robustness, we also used quantile regression to model the 25th and 75th percentiles of ASPQ scores.

We also performed a supplementary univariable quantile regression analysis to estimate the unadjusted association of each demographic variable with the median APSQ total score and the subscale scores.

### 2.6 Population norm values and summary of the APSQ scores

Population norms were derived for the median, 25th and 75th percentile of APSQ scores, stratified for all factors that were significantly associated with changes in the APSQ score. To calculate norm values, we predicted average values for each stratification level (estimated marginal means) based on the fitted quantile regression models estimating the median APSQ scores and their 25th and 75th percentiles. In addition, we provided descriptive summaries of APSQ scores. Scores were described in terms of absolute number with percentages or with medians and interquartile ranges (IQR); percentiles (0th−100th) were calculated for the total and subscale scores across the entire cohort.

### 2.7 Factors associated with daily usage duration

Multivariable ordinal logistic regression models were used to assess the association of APSQ scores and device and demographic factors with changes in daily usage duration. Four separate models were fit, one including the total score and one each for the subscale scores. Each model included five main effects: APSQ score, audio processor type, hearing configuration, gender, and age. No interaction effects were specified. Generalized VIF were all below a value of 1.12.

The proportional odds assumption was evaluated using graphical methods and formal tests comparing the proportional odds model with partial proportional odds and multinomial alternatives. As model fit did not improve with either alternative (lower Akaike information criterion and non-significant χ^2^ tests), the proportional odds model was retained. Model fitting used the R package VGAM (“vector generalized linear and additive models”; Yee, 2025), and proportional odds diagnostics followed procedures implemented in the R package rms (Harrell, 2025) as described by Harrell (2015).

As self-reported daily usage duration was assessed in 3 h steps (*0–3, 3–6*, 6–9, 9–12, and >*12 h*), the models estimated the log odds of participants using their CI at least 3 h less in a day. For reporting, positive log odds coefficients were transformed to odds ratios (OR), quantifying the odds of a ≥3 h decrease in daily usage duration relative to the reference factor level. For reporting negative log odds coefficients, the reciprocal of the odds ratio was taken (indicated in the text as *inverse* OR). This inversion was used to facilitate interpretation, as all effect sizes are expressed as values greater than one. Inverse ORs correspond to the odds of a ≥3 h increase in daily usage duration relative to the reference factor level.

We also performed a supplementary univariable ordinal logistic regression analysis to report the unadjusted association between the factor and the duration of the daily usage duration.

### 2.8 Item-level analysis

We hypothesized that some item scores might differ between OTE and BTE processors. To assess this, we compared the distributions of scores for the following six items:

*It is easy to put the AP on its proper place on my head* (*usability*, Q2).

*My AP is skin-friendly* (*wearing comfort*, Q3).

*My AP is comfortable to wear* (*wearing comfort*, Q6).

*I can comfortably wear glasses and my AP at the same time* (*wearing comfort*, Q9).

*I can comfortably wear headwear and my AP at the same time* (*wearing comfort*, Q12).

*My AP stays in the same position all day* (*wearing comfort*, Q15).

We excluded participants who did not provide responses to all six of these questions. This yielded unbalanced data: 562 responses from BTE users and 211 responses from OTE users.

The Mann–Whitney *U* test was used to compare scores. Effect sizes were estimated with Pearson's *r*. Due to the large sample sizes, only comparisons which were both statistically significant and with Pearson's *r* > 0.1 were regarded as genuine effects.

## 3 Results

### 3.1 Cohort description and overall APSQ scores

The distributions of demographic and device characteristics are shown in Table 1. Most characteristics were relatively equally distributed between the two audio processor types. However, female participants used OTE processors more frequently than male participants (35% vs. 21%). Respondents with SSD used BTE processors less frequently than those with bilateral CIs (60% vs. 82%).

**Table 1:** Participant characteristics, including stratification by audio processor type.

Condition	Overall n (column %)	BTE n (row %)	OTE n (row %)
Total participants/ears	861	620 (*72%*)	241 (*28%*)
Gender			
Female	435 (51%)	284 (*65%*)	151 (*35%*)
Male	415 (48%)	328 (*79%*)	87 (*21%*)
Other^†^	11 (1.3%)	8 (*73%*)	3 (*27%*)
Hearing configuration			
Bilateral CI	307 (36%)	253 (*82%*)	54 (*18%*)
Unilateral CI	130 (15%)	91 (*70%*)	39 (*30%*)
Bimodal	255 (30%)	174 (*68%*)	81 (*32%*)
SSD	169 (20%)	102 (*60%*)	67 (*40%*)
Age categories			
< 5	1 (0.1%)	1 (*100%*)	0 (*0%*)
≥5 to < 18	16 (1.9%)	13 (*81%*)	3 (*19%*)
≥18 to < 40	107 (12%)	82 (*77%*)	25 (*23%*)
≥40 to < 60	301 (35%)	200 (*66%*)	101 (*34%*)
≥60 to < 80	391 (45%)	291 (*74%*)	100 (*26%*)
≥80	45 (5.2%)	33 (*73%*)	12 (*27%*)
Daily usage duration			
>12 h	641 (74%)	475 (*74%*)	166 (*26%*)
9–12 h	153 (18%)	99 (*65%*)	54 (*35%*)
6–9 h	47 (5.5%)	31 (*66%*)	16 (*34%*)
3–6 h	14 (1.6%)	12 (*86%*)	2 (*14%*)
0–3 h^*^	6 (0.7%)	3 (*50%*)	3 (*50%*)
Age in years	60 (50, 69)	60 (50, 70)	59 (51, 67)

All values are numbers with percentages (row percentages in italics). Age in years is given with the median and IQR.

^*^The options never and 0–3 h were combined for inferential analysis.

^†^This group was excluded from inferential analysis due to the small sample size.

**Figure 2:**
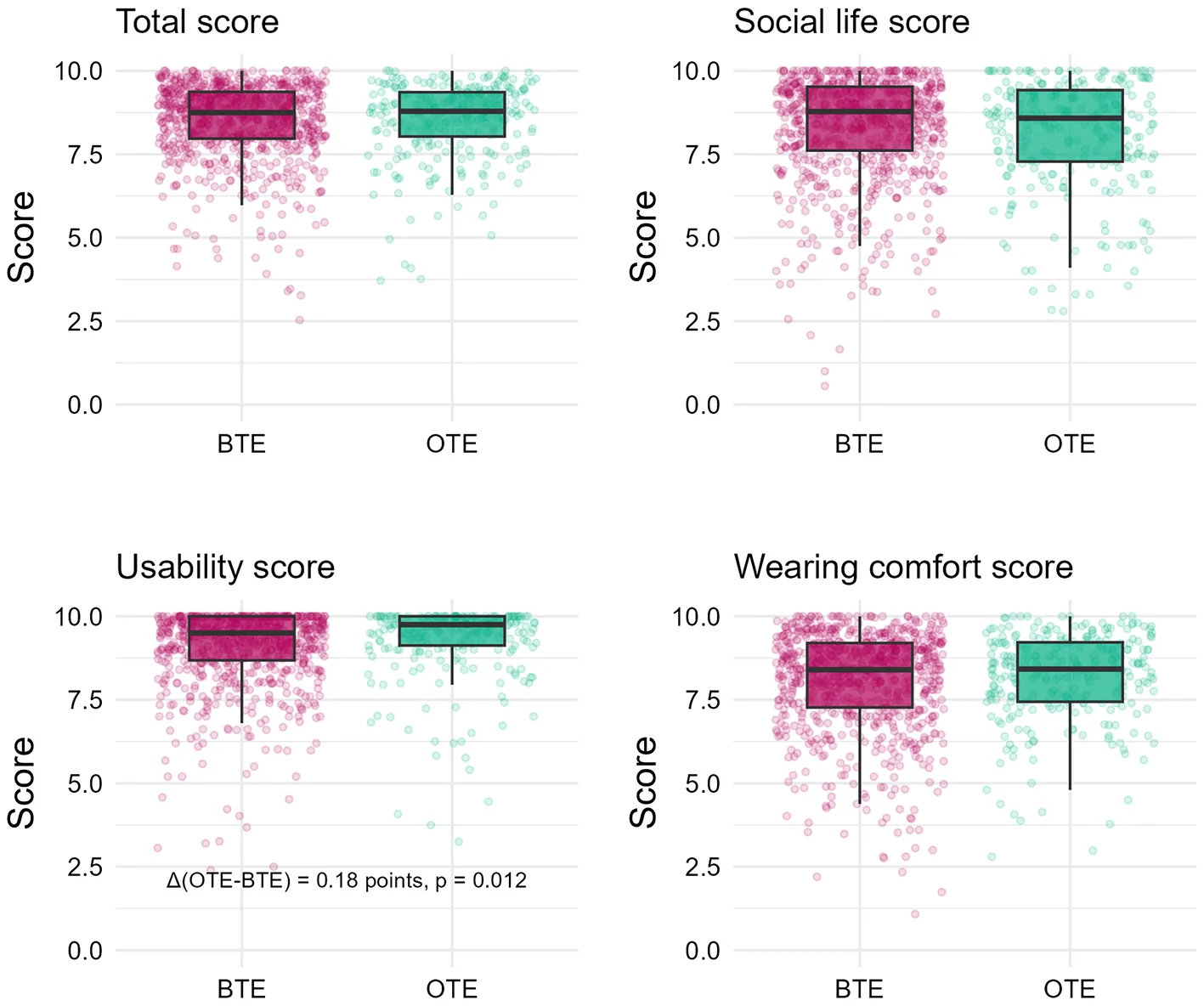
Distributions of APSQ scores stratified by audio processor type. Significant effects of processor type are labeled.

**Figure 3:**
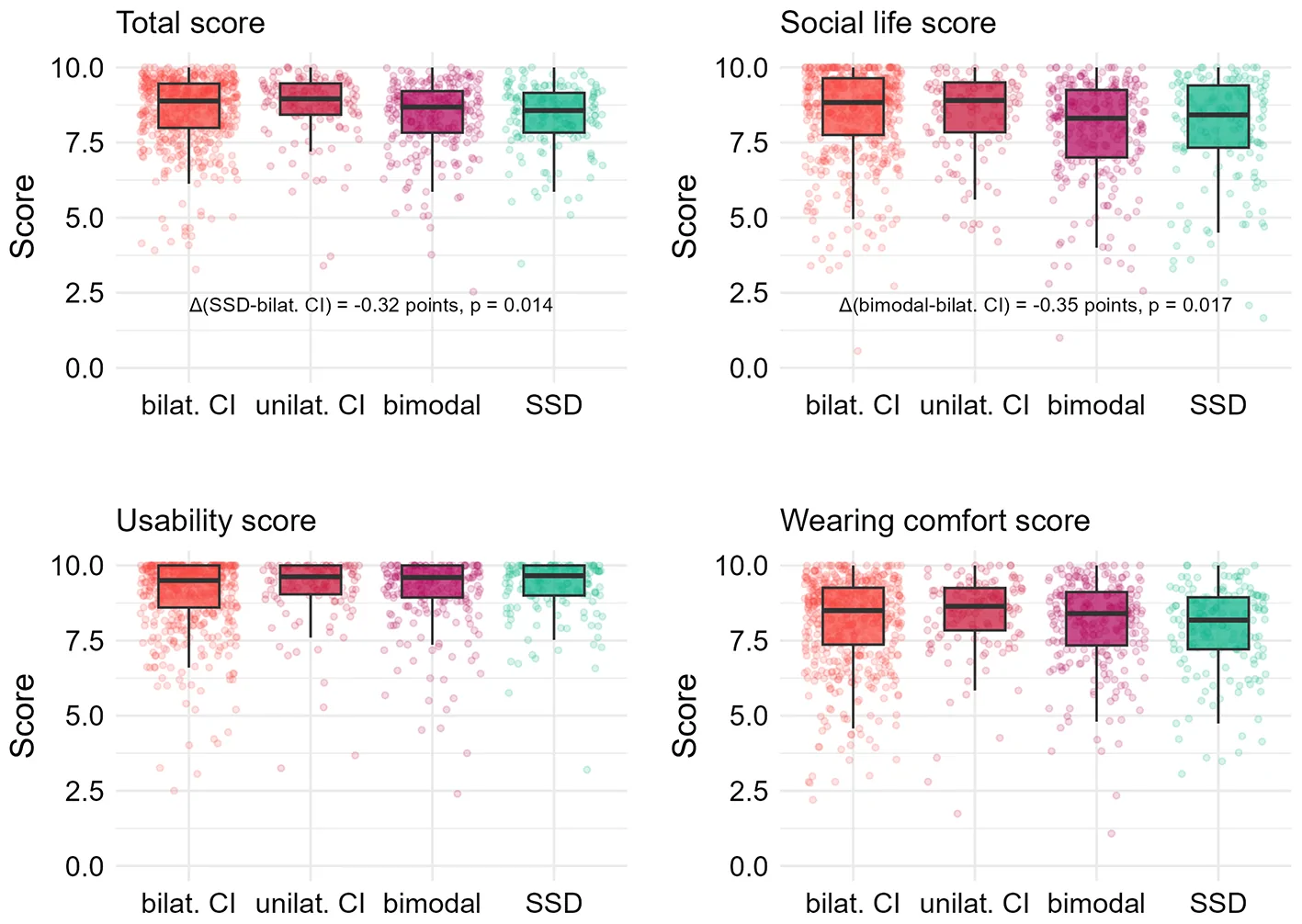
Distributions of APSQ scores stratified by hearing configuration. Hearing configurations with significant effects relative to bilateral CI are labeled.

Table 2 shows the distributions of total and subscale scores, stratified by gender, hearing configuration, age category, and audio processor types. The score distributions were relatively similar across most categories. The outliers were those which had small sample sizes (marked with an asterisk). The distributions of scores are shown stratified by AP type and by hearing configuration in Figures 2, 3.

**Table 2:** APSQ total and subscale scores, stratified by participant characteristics.

Condition	Total	Social life	Usability	Comfort
Overall	8.7 (7.9, 9.3)	8.6 (7.4, 9.4)	9.6 (8.9, 10.0)	8.4 (7.4, 9.2)
Gender				
Female	8.8 (8.1, 9.4)	8.9 (7.7, 9.6)	9.6 (9.0, 10.0)	8.4 (7.4, 9.2)
Male	8.6 (7.8, 9.3)	8.4 (7.0, 9.4)	9.5 (8.7, 10.0)	8.4 (7.3, 9.1)
Other^*^	8.0 (7.7, 9.4)	8.2 (7.0, 9.3)	9.1 (8.3, 9.5)	7.8 (7.2, 9.4)
Hearing configuration				
Bilateral CI	8.9 (8.0, 9.5)	8.9 (7.8, 9.6)	9.5 (8.6, 10.0)	8.4 (7.3, 9.2)
Unilateral CI	8.9 (8.4, 9.5)	8.9 (7.8, 9.5)	9.6 (9.0, 10.0)	8.6 (7.8, 9.3)
Bimodal	8.7 (7.8, 9.2)	8.3 (7.0, 9.3)	9.6 (8.9, 10.0)	8.4 (7.3, 9.1)
SSD	8.6 (7.8, 9.2)	8.4 (7.3, 9.4)	9.6 (9.0, 10.0)	8.2 (7.2, 8.9)
Age categories				
< 5^*^	9.8 (9.8, 9.8)	10.0 (10.0, 10.0)	10.0 (10.0, 10.0)	9.3 (9.3, 9.3)
≥5 to < 18^*^	8.5 (8.1, 9.3)	9.0 (8.4, 9.5)	9.4 (8.6, 9.9)	8.0 (7.3, 9.0)
≥18 to < 40	8.6 (7.6, 9.2)	8.8 (7.6, 9.5)	9.4 (8.4, 9.9)	8.1 (7.0, 8.8)
≥40 to < 60	8.7 (8.0, 9.3)	8.8 (7.5, 9.5)	9.6 (9.0, 10.0)	8.3 (7.3, 9.1)
≥60 to < 80	8.8 (8.0, 9.4)	8.6 (7.3, 9.4)	9.6 (8.9, 10.0)	8.6 (7.5, 9.3)
≥80	8.6 (8.0, 9.2)	8.1 (7.1, 8.9)	9.3 (8.8, 10.0)	8.5 (7.6, 9.1)
Audio processor type				
BTE	8.7 (8.0, 9.4)	8.7 (7.5, 9.5)	9.5 (8.8, 10.0)	8.4 (7.3, 9.2)
OTE	8.7 (7.9, 9.3)	8.4 (7.3, 9.4)	9.8 (9.1, 10.0)	8.4 (7.4, 9.2)

The values are medians with the IQR.

^*^These groups had small sample sizes (*n* = 1–16).

### 3.2 Factors associated with APSQ scores

The estimated marginal effects on median APSQ scores are given in Table 3. The factors used in this model explained only a small amount of variance in the APSQ scores: pseudo *R*^2^ values were 0.017 for the total score, 0.031 for *social life*, 0.011 for *usability*, and 0.016 for *wearing time*. Overall, effects were small, not exceeding 0.35 points on the ASPQ scale.

**Table 3:** Marginal effects on median APSQ scores estimated with multivariable quantile regression.

Condition	Total	Social life	Usability	Comfort
(Intercept)	8.93 (8.78, 9.08)	8.96 (8.74, 9.18)	9.57 (9.43, 9.71)	8.49 (8.25, 8.72)
	< 0.001	< 0.001	< 0.001	< 0.001
Audio processor type				
OTE	−0.02 (−0.20, 0.15)	−0.28 (−0.57, 0.01)	**0.18 (0.04, 0.32)**	0.02 (−0.20, 0.25)
	0.807	0.055	**0.012**	0.852
Hearing configuration				
Unilateral CI	0.11 (−0.12, 0.34)	0.10 (−0.20, 0.39)	0.06 (−0.16, 0.27)	0.21 (−0.08, 0.50)
	0.359	0.514	0.611	0.16
Bimodal	−0.18 (−0.38, 0.02)	**−0.35 (−0.64**, **−0.06)**	0.04 (−0.14, 0.22)	−0.13 (−0.40, 0.15)
	0.085	**0.017**	0.686	0.372
SSD	**−0.32 (−0.58**, **−0.07)**	−0.24 (−0.59, 0.11)	0.06 (−0.14, 0.26)	−0.31 (−0.66, 0.04)
	**0.014**	0.176	0.561	0.082
Gender				
Male	**−0.20 (−0.38**, **−0.02)**	**−0.30 (−0.55**, **−0.05)**	−0.14 (−0.28, 0.01)	−0.13 (−0.35, 0.09)
	**0.027**	**0.018**	0.062	0.234
Age				
Per 10-year increase	0.05 (−0.01, 0.11)	**−0.07 (−0.14**, **−0.00)**	0.01 (−0.04, 0.06)	**0.11 (0.04, 0.18)**
	0.087	**0.048**	0.696	**0.002**

Estimates with 95% confidence intervals are given above. *p*-values are given below. Significant factors are given in bold. The reference factor levels were BTE for audio processor, bilateral CI for hearing configuration, female for gender, and mean age (57 years) for age (APSQ scores for this combination estimated by the intercept). Age estimates are given per 10-year increase beyond the mean age. Standard errors were estimated via bootstrapping.

The audio processor type was associated with a small but statistically significant change on the *usability* subscale score, with slightly higher scores for OTE processors (difference: 0.18 points, *p* = 0.012; Figure 2).

For hearing configuration, bimodal users had a slightly lower median score on the *social life* subscale compared to bilateral CI users (−0.35 points, *p* = 0.017). SSD users had slightly lower total scores compared to bilateral CI users (−0.32 points, *p* = 0.014; Figure 3).

Male gender was associated with slightly lower total scores (−0.20 points, *p* = 0.027) and *social life* subscale scores (−0.30 points, *p* = 0.018). There were significant effects of age on the *social life* and *wearing comfort* subscale scores, but all of these were negligible in size.

The adjusted estimates were similar to those observed via univariable regression (Supplementary Table 1) and multivariable regression modeling the 25th and 75th percentile (Supplementary Tables 2, 3).

### 3.3 Population norm values

To facilitate the interpretation of individual APSQ scores, we provided a table of the percentiles for the total score and subscale scores (Table 4). A ceiling effect was evident for the APSQ total score and all three subscales. Nevertheless, scores showed a clear linear increase between the 10th and 90th percentiles for the total score and the *social life* and *wearing comfort* subscales, and between the 10th and 70th percentiles for the *usability* subscale.

**Table 4:** Percentiles of APSQ total and subscale scores.

Percentile	Total	Social life	Usability	Comfort
0	2.5	0.6	2.4	1.1
10	7.0	6.1	7.8	6.2
20	7.8	7.0	8.6	7.1
30	8.1	7.8	9.0	7.6
40	8.5	8.3	9.3	8.0
50	8.7	8.6	9.6	8.4
60	9.0	9.0	9.8	8.7
70	9.2	9.3	10.0	9.0
80	9.5	9.6	10.0	9.3
90	9.7	10.0	10.0	9.7
100	10.0	10.0	10.0	10.0

These were computed over the entire cohort (*n* = 861). Note that reference scores may not be representative for groups which significantly deviate from the average or for groups for with a small sample size.

While scores covered almost the whole 10-point APSQ scale (see the 0th and 100th percentile in Table 4 marking the minimum and maximum scores), only few scores—less than 10—below 7 points on the 10-point APSQ scale were observed for the total scores and subscales. A ceiling of 10 points was reached around the 90th percentile for the total score and the *social life* and *wearing comfort* subscales and around the 70th percentile for the *usability* subscale.

As some factors were significantly associated with APSQ scores, we also stratified population norm values by these factors to allow for a more detailed assessment of individual APSQ scores. These were hearing configuration and gender for the total and *social life* scores (Supplementary Figure 1 and Supplementary Table 4), AP type for the *usability* scores (Supplementary Figure 2 and Supplementary Table 5) and age for the *wearing comfort* scores (Supplementary Figure 3 and Supplementary Table 6).

### 3.4 Factors associated with daily usage duration

**Figure 4:**
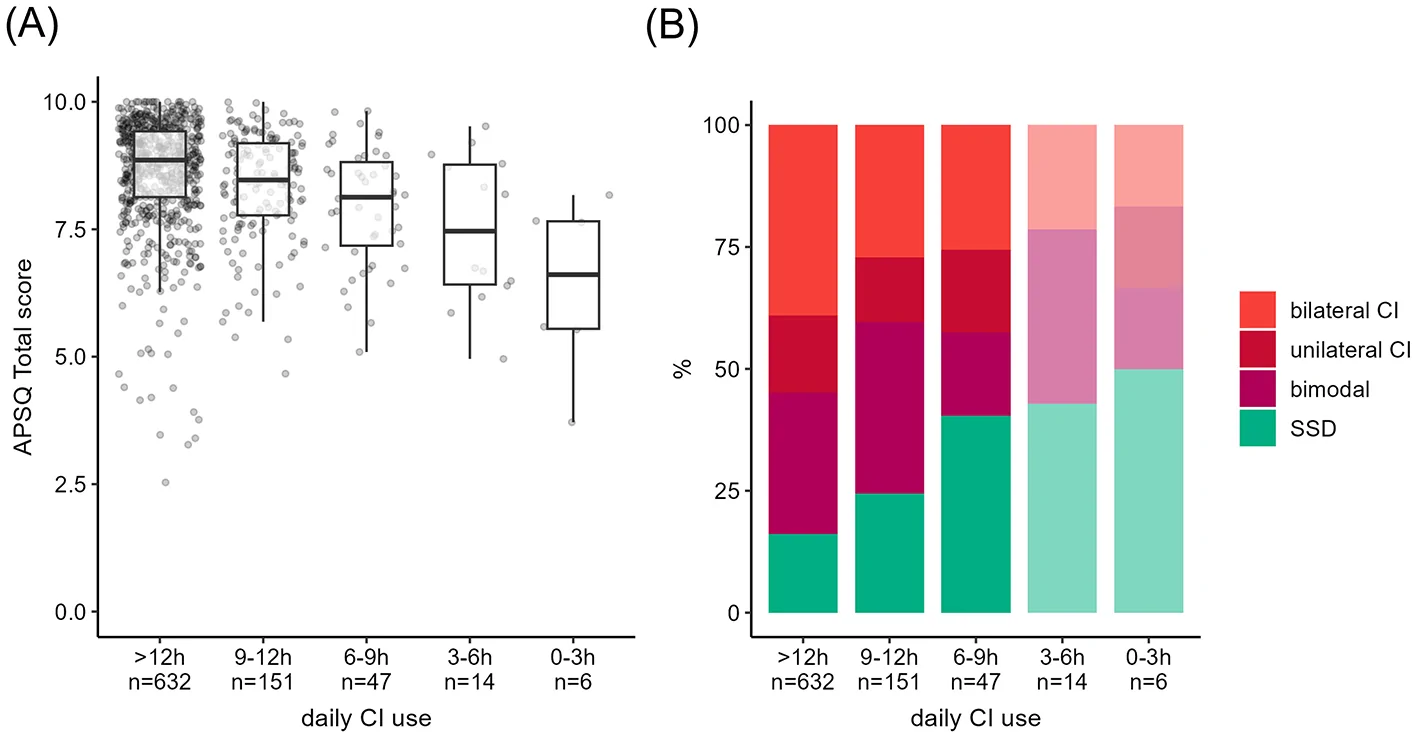
**(A)** Distribution of APSQ total scores by daily usage duration category. **(B)** Distribution of usage duration categories stratified by hearing configuration. Note the few responses for the categories 0–3 h and 3–6 h.

Daily usage duration was generally high. 76% of participants reported using their audio processor for more than 12 h per day (Table 1). The average total score increased monotonically with usage duration (Figure 4A).

**Figure 5:**
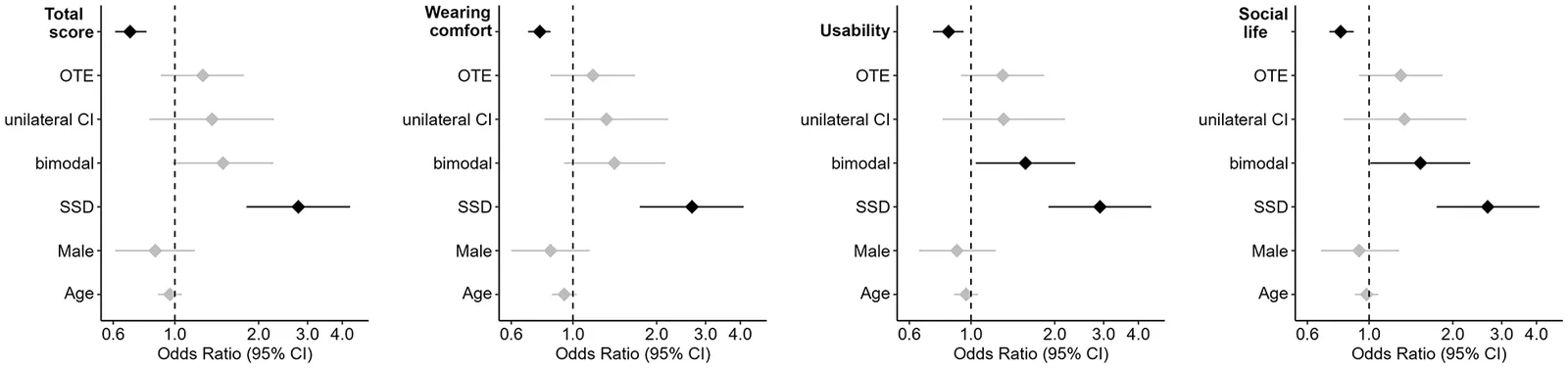
Odds ratios representing an increased risk for the lowest daily usage duration category. Significant estimates are marked in black.

This association was corroborated by the multivariable ordinal logistic regression model (Figure 5, Supplementary Table 7). For each additional point on the total score, the odds of using the CI for 3 h longer were approximately 44% higher (inverse OR = 1.44; 95% CI: 1.27, 1.63; *p* < 0.001). Similar effects were observed for the subscale score models.

Compared to bilateral CI users, SSD CI users had significantly higher odds of using their CI on average 3 h less per day (OR = 2.78 = 178% higher odds for shorter daily usage duration; 95% CI: 1.81–4.27; *p* < 0.001; Figures 4B, 5). Similar effects were observed for the subscale score models.

No other demographic factor was independently associated with an altered risk of device daily usage duration in the model that included total APSQ scores. However, in the *usability* and *wearing comfort* models, bimodal users had significantly higher odds than bilateral CI users of lower device usage (*usability*: OR = 1.57; 95% CI: 1.04–2.37; *p* = 0.032; *wearing comfor*t: OR = 1.53; 95% CI: 1.01–2.31; *p* = 0.046).

Univariable analysis yielded odds ratios that were very similar to those from multivariable analysis (Supplementary Table 8). The only major difference was that OTE devices were associated with a small increase of daily usage duration relative to BTE devices.

### 3.5 Item-level analysis

**Figure 6:**
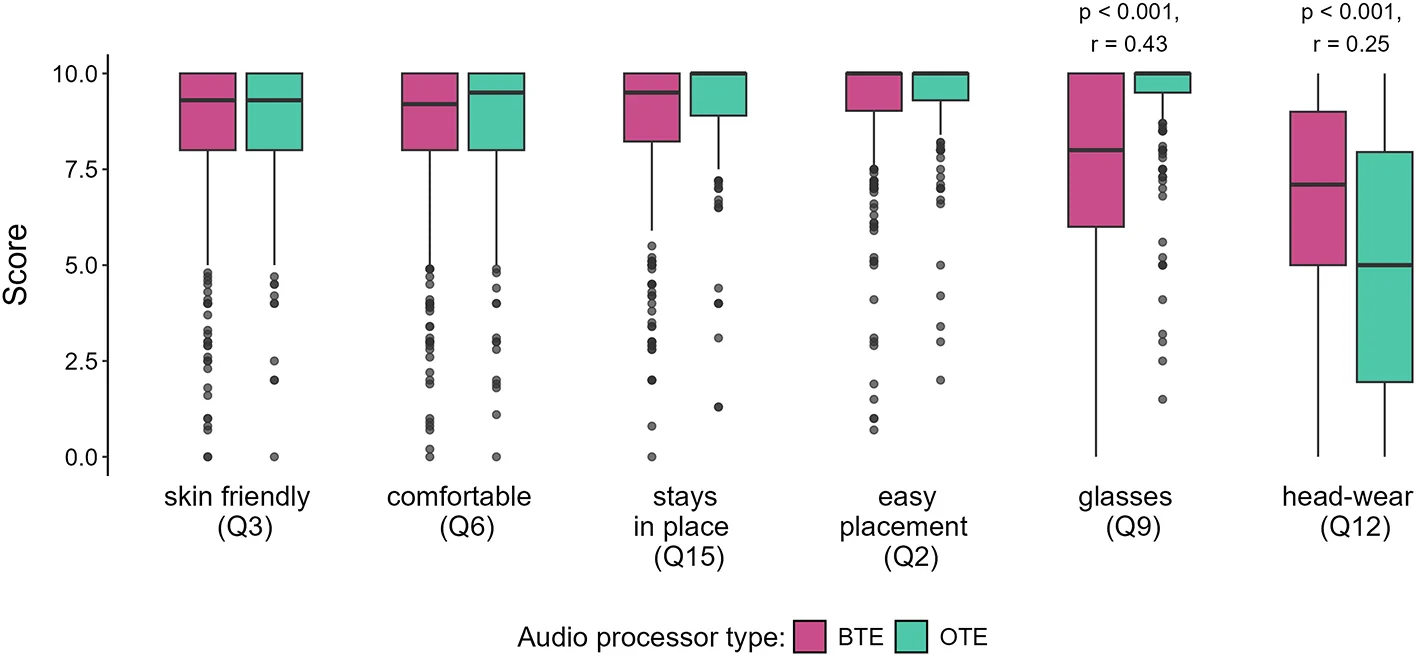
Distributions of scores on the six items of interest between the BTE and OTE processors. Significant effects of processor types are labeled.

The distributions of responses to the six items of comparison between BTE and OTE processors are shown in Figure 6.

Users of OTE processors reported higher satisfaction with their ability to comfortably wear glasses (median 10.0 vs. 8.0, *r* = 0.43, *p* < 0.001). Users of BTE processors had higher satisfaction with their ability to comfortably wear headwear (median 7.3 vs. 5.2, *r* = 0.25, *p* < 0.001).

No significant differences were observed between scores for the other four items of interest.

## 4 Discussion

### 4.1 Factors associated with APSQ scores

We found several factors that were associated with APSQ total and subscale scores. However, the magnitudes of these effects were small in all cases, never exceeding 0.35 points (on a 0–10 scale). This is lower than the test-retest variability of the German APSQ (roughly 0.56 points, Billinger-Finke et al., 2020). Nevertheless, these apparent effects are discussed below.

Total scores were negatively influenced by SSD and male gender with respect to their reference classes. It has been observed that CI users with SSD often have lower rates of daily usage duration than bilateral CI users (Lin et al., 2025). With SSD, the user has a contralateral normal hearing ear, which reduces reliance upon the CI and can also create a mismatched auditory image. This may explain the lower rates of satisfaction among SSD users. Note however that APSQ scores were still quite high in this group; the median total score was 8.6, and 75% of users reported scores above 7.8 (Table 2).

The lower total scores for males is somewhat harder to explain. This effect was very small; on average males rated their total satisfaction 0.2 points lower than females. No gender difference is evident in the data provided in two prior studies with the APSQ (Felder et al., 2022, Supplementary material 2 and Alasmi et al., 2024, Supplementary material 3).

No other characteristics were associated with total scores. For the subscale scores, some significant effects were observed; their effect sizes ranged in magnitude from 0.07 to 0.35. We regard these effects as marginal, both for their small size and because these effects did not impact the total satisfaction score.

### 4.2 Population norm values

We used our large reference cohort to derive population norm values for the APSQ (Table 4 and Supplementary Tables 4–6). As data collection was restricted to CI users in German- and English-speaking countries, strictly speaking these norm values apply only to these areas.

The validation study of the Arabic version of the APSQ found that the cumulative distributions of scores were quite similar across the German, English, and Arabic versions, whose study populations were drawn from Germany, the United States, and Saudi Arabia, respectively (Alasmi et al., 2024).

Given the relatively straightforward and practical nature of the items (for example Q2: “*It is easy to put the processor back on its proper place on my head*”) we posit that this instrument should be relatively resistant to cultural influences and semantic drift from translation.

### 4.3 Factors associated with daily usage duration

Taken together, our findings indicate that AP satisfaction was associated with daily usage duration. AP type, gender, and age had no independent association with daily usage duration when satisfaction scores were included in the models. We emphasize that these are statistical associations only; we do not assert any causal relationship between these variables based on the findings presented here.

In the model containing total APSQ scores, only users with SSD had an independent association with lower daily usage duration (relative to bilateral CI users). Bimodal users had an independent association with lower daily usage duration, but only within models containing the *usability* or *wearing comfort* scores. The most parsimonious explanation is that bimodal users experience challenges that are related to practical and comfort issues but not related to their social life. In comparison, SSD users exhibit more global challenges, including in their social life.

Although we found an association between satisfaction and daily usage duration, we cannot determine the direction of causality, if a causal relationship exists at all. Does higher device use lead to greater satisfaction, or are satisfied users more inclined to use their device? Answering this would require an interventional study where users are assigned to a higher level of daily usage duration. This is beyond the scope of the present study.

### 4.4 Item-level analysis

Here we found that scores on two items differed significantly between BTE and OTE processors. These were for items related to wearing of glasses (OTE median 10.0 vs. BTE median 8.0) and headwear (OTE median 5.2 vs. BTE median 7.3). These findings are straightforward to interpret, given the relative head positioning of the different AP types. A very similar trend is evident in the data of the English APSQ validation study (Felder et al., 2022, Supplementary material 2).

For both processor types, satisfaction was on average higher for the ability to wear glasses compared to headwear. The gap was much wider with OTE devices, where few experienced issues with their glasses, while lower satisfaction with headwear was relatively common.

### 4.5 Ceiling effect

As observed previously and confirmed here, the APSQ has a ceiling effect (Billinger-Finke et al., 2020). According to the present data, this limits discrimination between users with scores higher than 90% of the population (or higher than 70% of the population for the usability subscale). The questionnaire items were not designed to bias respondents toward higher satisfaction. The items were designed to be focused and practical (for example Q8: “*It is easy to switch the processor on and off* ”). The ceiling effect nevertheless warrants explanation.

CI processors are medical devices which are intended to be used during all waking h. This requires initiative and motivation on the part of the user. As such, much consideration goes into the design of these devices to ensure they are comfortable and easy to use. These designs have been iteratively refined over several decades in a competitive industry. In light of this, it is perhaps not surprising that the typical user satisfaction level is high. Nevertheless, we also observed instances of low scores, meaning the instrument may be useful to detect cases where satisfaction is substantially lower than average.

The practical limitation of this ceiling effect is that it is difficult to distinguish between a user who is quite satisfied and one who is very satisfied. Despite this, the present study found that responses are generally linearly increasing between the 10th and the 70th/90th percentiles, which suggests that the questionnaire can distinguish between a large range of satisfaction levels. In addition, prior studies have shown that the APSQ has the sensitivity to detect improvements in satisfaction after an AP upgrade (Lailach et al., 2023), longitudinal improvements with same AP (Zernotti et al., 2021; Lassaletta et al., 2022; Urík et al., 2022), and differences in satisfaction between BTE and OTE type APs (Urík et al., 2022).

### 4.6 Potential for response bias

The survey was conducted on a voluntary basis. Users completed and returned the questionnaire on their own initiative. This introduces the risk of a response bias where only those with strong feelings about their device are inclined to complete the survey. Nevertheless, the distributions of scores here were quite similar to those found in the prior validation studies, where participants were recruited for the specific purpose of completing the questionnaire (Billinger-Finke et al., 2020; Felder et al., 2022; Alasmi et al., 2024).

In this study users were required to have at least one month of experience with the device. This lower-bound cutoff was a pragmatic choice to ensure compliance with a voluntary questionnaire. Requiring a longer duration may have reduced compliance. Potentially, this could introduce response biases if users have not fully acclimated to their device by the time they complete the questionnaire. However, as stated above, the response distributions were similar to those in a prior validation study where users had longer durations of experience (mean 4.7 years, range: 3 months−16 years; Felder et al., 2022).

In addition, it has been reported that CI users may tend to over-estimate their self-reported device usage relative to objective datalogging. For example, Holder et al. (2022) reported an average discrepancy of 2.6 h/day. Due to the anonymous nature of the questionnaire, we could not pair the self-reported usage data with objective datalogging records. This is a limitation of our study. The main impact of overestimation, if present in our data, is that some users were pushed into a higher usage category than they would have been if datalogging records were used instead.

## 5 Conclusion

The APSQ is a robust and practical tool for quantifying audio processor satisfaction. The population norm values derived here are useful to guide clinical interpretation. Our findings demonstrate that subjective satisfaction is a primary determinant of daily usage duration, offering valuable insights for improving long–term user engagement and device adherence.

## Data Availability

The raw data supporting the conclusions of this article will be made available by the authors, without undue reservation.
