# Supplementary material for: User satisfaction with cochlear implant audio processors: population norms derived from a large-scale survey campaign
Source: Front Psychol. 2026 Sep 7;17:1817444. doi: 10.3389/fpsyg.2026.1817444 (PMC13590707; doi:10.3389/fpsyg.2026.1817444)
Supplement: Supplementary file 1 [file Data_Sheet_1.docx]

**Supplementary Information**

**Supplementary Figures**

**Supplementary Figure 1:** APSQ population norm values stratified by hearing configuration and by gender for all ages, as estimated with quantile regression. The dots show the estimates of the 25^th^, 50^th^, 75^th^ percentiles. The bars show the 95% confidence intervals of the estimates.

**Supplementary Figure 2:** APSQ usability population norm values by audio processor type over all hearing configurations, ages and both genders. The dots show the estimates of the 25^th^, 50^th^, and 75^th^ percentiles. The bars show the 95% confidence intervals of the estimates.

**Supplementary Figure 3:** APSQ wearing comfort population norm values by users age over all hearing configurations and both genders. The estimates of the 25^th^, 50^th^, and 75^th^ percentiles are shown. The bands show the 95% confidence intervals of the estimates.

**Supplementary Tables**

**Supplementary Table 1**: Effects on median APSQ scores estimated with univariable quantile regression. Estimates with 95% confidence intervals are given above. *p*-values are given below. Significant predictors are given in bold. The reference predictor levels were BTE for audio processor, bilateral CI for hearing configuration, female for gender, and mean age for age. Per decade indicates an additional decade of age beyond the mean age. Standard errors were estimated via bootstrapping. *The intercepts varied per model.

|  |  | **Total** | **Social life** | **Usability** | **Comfort** |
| --- | --- | --- | --- | --- | --- |
| **(Intercept)*** |  | – | – | – | – |
| **Audio processor type** | OTE | -0.00 (-0.16, 0.16) | -0.28 (-0.56, 0.00) | **0.25 (0.13, 0.37)** | -0.00 (-0.20, 0.20) |
|  |  | .963 | .054 | ***<.001*** | 1.000 |
| **Hearing configuration** | Unilateral CI | 0.10 (-0.16, 0.35) | 0.08 (-0.22, 0.38) | 0.11 (-0.11, 0.32) | 0.20 (-0.11, 0.51) |
|  |  | .465 | 0.596 | 0.334 | .206 |
|  | Bimodal | -0.17 (-0.39, 0.04) | **-0.50 (-0.77, -0.23)** | 0.08 (-0.08, 0.24) | -0.04 (-0.31, 0.23) |
|  |  | .115 | ***<0.001*** | 0.323 | .774 |
|  | SSD | **-0.29 (-0.55, -0.04)** | **-0.40 (-0.71, -0.09)** | 0.14 (-0.08, 0.36) | -0.26 (-0.60, 0.08) |
|  |  | ***.026*** | ***0.011*** | 0.219 | .140 |
| **Gender** | Male | -0.17 (-0.36, 0.03) | **-0.48 (-0.70, -0.26)** | -0.12 (-0.28, 0.03) | -0.00 (-0.22, 0.22) |
|  |  | .092 | ***<.001*** | .118 | 1.000 |
| **Age** | Per decade | 0.01 (-0.04, 0.07) | **-0.09 (-0.16, -0.02)** | 0.01 (-0.04, 0.05) | **0.10 (0.03, 0.18)** |
|  |  | .671 | **.013** | .757 | **.006** |

**Supplementary Table 2**: Effects on 25^th^ percentile of APSQ scores estimated with multivariable quantile regression. Estimates with 95% confidence intervals are given above. *p*-values are given below. Significant predictors are given in bold. The reference predictor levels were BTE for audio processor, bilateral CI for hearing configuration, female for gender, and mean age for age. Per decade indicates an additional decade of age beyond the mean age. Standard errors were estimated via bootstrapping.

|  |  | **Total** | **Social life** | **Usability** | **Comfort** |
| --- | --- | --- | --- | --- | --- |
| **(Intercept)** |  | 8.09 (7.90, 8.27) | 8.08 (7.70, 8.47) | 8.70 (8.39, 9.01) | 7.41 (7.05, 7.76) |
|  |  | <0.001 | <0.001 | <0.001 | <0.001 |
| **Audio processor type** | OTE | -0.08 (-0.36, 0.20) | -0.39 (-0.82, 0.03) | **0.28 (0.01, 0.54)** | 0.13 (-0.25, 0.52) |
|  |  | 0.566 | 0.069 | **0.041** | 0.498 |
| **Hearing configuration** | Unilateral | **0.52 (0.18, 0.87)** | 0.17 (-0.42, 0.77) | **0.48 (0.09, 0.86)** | 0.34 (-0.14, 0.82) |
|  |  | **0.003** | 0.563 | **0.015** | 0.16 |
|  | Bimodal | -0.03 (-0.30, 0.24) | -0.31 (-0.85, 0.24) | 0.30 (-0.05, 0.65) | -0.17 (-0.61, 0.28) |
|  |  | 0.823 | 0.275 | 0.097 | 0.463 |
|  | SSD | -0.08 (-0.38, 0.21) | -0.45 (-0.97, 0.07) | 0.28 (-0.09, 0.64) | -0.20 (-0.68, 0.29) |
|  |  | 0.568 | 0.087 | 0.135 | 0.424 |
| **Gender** | Male | **-0.28 (-0.53, -0.02)** | **-0.73 (-1.25, -0.21)** | **-0.28 (-0.54, -0.01)** | -0.09 (-0.43, 0.26) |
|  |  | **0.035** | **0.006** | **0.039** | 0.624 |
| **Age** | Per decade | 0.04 (-0.02, 0.10) | -0.09 (-0.22, 0.03) | -0.00 (-0.08, 0.08) | **0.12 (0.01, 0.24)** |
|  |  | 0.164 | 0.143 | 1.000 | **0.032** |

**Supplementary Table 3**: Effects on 75^th^ percentile of APSQ scores estimated with multivariable quantile regression. Estimates with 95% confidence intervals are given above. *p*-values are given below. Significant predictors are given in bold. The reference predictor levels were BTE for audio processor, bilateral CI for hearing configuration, female for gender, and mean age for age. Per decade indicates an additional decade of age beyond the mean age. Standard errors were estimated via bootstrapping.

|  |  | **Total** | **Social life** | **Usability** | **Comfort** |
| --- | --- | --- | --- | --- | --- |
| **(Intercept)** |  | 9.53 (9.38, 9.68) | 9.73 (9.54, 9.92) | 10.00 (9.96, 10.04) | 9.37 (9.14, 9.60) |
|  |  | <0.001 | <0.001 | <0.001 | <0.001 |
| **Audio processor type** | OTE | -0.07 (-0.22, 0.08) | -0.05 (-0.27, 0.17) | 0.00 (-0.02, 0.02) | -0.07 (-0.31, 0.17) |
|  |  | 0.344 | 0.633 | 1.000 | 0.555 |
| **Hearing configuration** | Unilateral | -0.00 (-0.19, 0.19) | 0.02 (-0.23, 0.28) | 0.00 (-0.04, 0.04) | -0.05 (-0.33, 0.23) |
|  |  | 0.975 | 0.863 | 1.000 | 0.724 |
|  | Bimodal | **-0.24 (-0.45, -0.04)** | **-0.36 (-0.62, -0.11)** | 0.00 (-0.04, 0.04) | -0.18 (-0.46, 0.09) |
|  |  | **0.022** | **0.004** | 1.000 | 0.188 |
|  | SSD | **-0.31 (-0.52, -0.11)** | **-0.31 (-0.60, -0.01)** | 0.00 (-0.06, 0.06) | **-0.30 (-0.60, -0.00)** |
|  |  | **0.003** | **0.043** | 1.000 | **0.047** |
| **Gender** | Male | -0.12 (-0.26, 0.02) | **-0.27 (-0.51, -0.04)** | -0.00 (-0.03, 0.03) | -0.16 (-0.37, 0.05) |
|  |  | 0.095 | **0.022** | 1.000 | 0.145 |
| **Age** | Per decade | 0.01 (-0.03, 0.05) | -0.03 (-0.08, 0.02) | -0.00 (-0.00, 0.00) | **0.08 (0.01, 0.15)** |
|  |  | 0.668 | 0.183 | 1.000 | **0.028** |

**Supplementary Table 4**: APSQ population norm values by hearing configuration and gender for all ages as estimated with quantile regression (median, 25^th^, 50^th^, and 75^th^ percentiles). Estimates are given with the 95% confidence interval in parentheses.

| **APSQ score** | **Gender** | **Percentile** | **Bilateral CI** | **Unilateral CI** | **Bimodal** | **SSD** |
| --- | --- | --- | --- | --- | --- | --- |
| Total | Female | 25% | 8.05 (7.85, 8.24) | 8.57 (8.26, 8.88) | 8.02 (7.76, 8.27) | 7.96 (7.68, 8.25) |
|  |  | 50% | 8.92 (8.76, 9.08) | 9.03 (8.84, 9.22) | 8.74 (8.58, 8.90) | 8.60 (8.40, 8.79) |
|  |  | 75% | 9.49 (9.36, 9.63) | 9.49 (9.33, 9.64) | 9.25 (9.06, 9.44) | 9.18 (9.00, 9.36) |
|  | Male | 25% | 7.77 (7.51, 8.03) | 8.30 (7.94, 8.65) | 7.74 (7.51, 7.97) | 7.69 (7.42, 7.96) |
|  |  | 50% | 8.72 (8.53, 8.91) | 8.83 (8.62, 9.04) | 8.54 (8.38, 8.71) | 8.40 (8.17, 8.62) |
|  |  | 75% | 9.37 (9.22, 9.53) | 9.37 (9.21, 9.53) | 9.13 (8.96, 9.30) | 9.06 (8.89, 9.23) |
|  |  |  |  |  |  |  |
|  |  |  |  |  |  |  |
|  |  |  |  |  |  |  |
| Comfort | Female | 25% | 7.48 (7.09, 7.87) | 7.82 (7.42, 8.22) | 7.31 (6.92, 7.71) | 7.28 (6.87, 7.69) |
|  |  | 50% | 8.50 (8.29, 8.72) | 8.71 (8.46, 8.97) | 8.38 (8.17, 8.59) | 8.19 (7.93, 8.45) |
|  |  | 75% | 9.33 (9.12, 9.55) | 9.28 (9.08, 9.49) | 9.15 (8.93, 9.37) | 9.03 (8.75, 9.32) |
|  | Male | 25% | 7.39 (6.99, 7.80) | 7.73 (7.30, 8.17) | 7.23 (6.84, 7.61) | 7.19 (6.80, 7.59) |
|  |  | 50% | 8.37 (8.11, 8.63) | 8.58 (8.31, 8.86) | 8.25 (8.01, 8.48) | 8.06 (7.78, 8.34) |
|  |  | 75% | 9.18 (8.97, 9.38) | 9.13 (8.92, 9.33) | 8.99 (8.77, 9.21) | 8.87 (8.59, 9.16) |
|  |  |  |  |  |  |  |
|  |  |  |  |  |  |  |
|  |  |  |  |  |  |  |
| Usability | Female | 25% | 8.84 (8.56, 9.12) | 9.31 (9.03, 9.59) | 9.14 (8.88, 9.40) | 9.11 (8.88, 9.34) |
|  |  | 50% | 9.66 (9.53, 9.79) | 9.71 (9.51, 9.92) | 9.69 (9.56, 9.82) | 9.72 (9.55, 9.88) |
|  |  | 75% | 10.00 (9.98, 10.02) | 10.00 (9.97, 10.03) | 10.00 (9.99, 10.01) | 10.00 (9.97, 10.03) |
|  | Male | 25% | 8.56 (8.21, 8.91) | 9.04 (8.74, 9.33) | 8.86 (8.62, 9.10) | 8.84 (8.55, 9.13) |
|  |  | 50% | 9.52 (9.35, 9.68) | 9.57 (9.38, 9.76) | 9.55 (9.39, 9.72) | 9.58 (9.41, 9.75) |
|  |  | 75% | 10.00 (9.96, 10.04) | 10.00 (9.96, 10.04) | 10.00 (9.97, 10.03) | 10.00 (9.95, 10.05) |
|  |  |  |  |  |  |  |
|  |  |  |  |  |  |  |
|  |  |  |  |  |  |  |
| Social life | Female | 25% | 7.88 (7.48, 8.28) | 8.05 (7.56, 8.55) | 7.57 (7.02, 8.12) | 7.42 (7.06, 7.79) |
|  |  | 50% | 8.82 (8.60, 9.03) | 8.91 (8.63, 9.19) | 8.46 (8.19, 8.73) | 8.57 (8.22, 8.92) |
|  |  | 75% | 9.70 (9.48, 9.92) | 9.72 (9.45, 9.99) | 9.33 (9.19, 9.48) | 9.39 (9.11, 9.67) |
|  | Male | 25% | 7.15 (6.67, 7.62) | 7.32 (6.75, 7.89) | 6.84 (6.43, 7.26) | 6.69 (6.08, 7.31) |
|  |  | 50% | 8.51 (8.25, 8.78) | 8.61 (8.30, 8.92) | 8.16 (7.90, 8.42) | 8.27 (7.92, 8.62) |
|  |  | 75% | 9.43 (9.19, 9.66) | 9.45 (9.22, 9.68) | 9.06 (8.84, 9.28) | 9.12 (8.78, 9.46) |

**Supplementary Table 5**: APSQ Usability population norm values by audio processor type over all hearing configurations, ages, and both genders. Median, 25^th^, 50^th^, and 75^th^ percentiles were estimated with quantile regression.

| **Audio processor type** | **Percentile** | **Estimate (95% CI)** |
| --- | --- | --- |
| BTE | 25% | 8.82 (8.66, 8.99) |
|  | 50% | 9.54 (9.44, 9.63) |
|  | 75% | 10.00 (9.97, 10.03) |
| OTE | 25% | 9.10 (8.88, 9.32) |
|  | 50% | 9.71 (9.60, 9.83) |
|  | 75% | 10.00 (9.98, 10.02) |

**Supplementary Table 6**: APSQ wearing comfort population norm values by age over all hearing configurations and both genders. The 25^th^, 50^th^, and 75^th^ percentiles were estimated with quantile regression.

|  | **Percentile** | | |
| --- | --- | --- | --- |
| **Age (years)** | **25^th^** | **50^th^** | **75^th^** |
| 10 | 6.85 (6.30, 7.39) | 7.85 (7.42, 8.29) | 8.76 (8.43, 9.09) |
| 15 | 6.91 (6.41, 7.40) | 7.91 (7.52, 8.30) | 8.80 (8.50, 9.09) |
| 20 | 6.97 (6.53, 7.41) | 7.97 (7.61, 8.32) | 8.83 (8.57, 9.10) |
| 25 | 7.03 (6.64, 7.42) | 8.02 (7.70, 8.34) | 8.87 (8.63, 9.11) |
| 30 | 7.09 (6.75, 7.43) | 8.08 (7.80, 8.36) | 8.91 (8.70, 9.12) |
| 35 | 7.15 (6.86, 7.45) | 8.13 (7.89, 8.37) | 8.95 (8.77, 9.13) |
| 40 | 7.22 (6.97, 7.46) | 8.19 (7.98, 8.39) | 8.99 (8.83, 9.15) |
| 45 | 7.28 (7.07, 7.49) | 8.24 (8.07, 8.42) | 9.03 (8.89, 9.16) |
| 50 | 7.34 (7.16, 7.52) | 8.30 (8.16, 8.44) | 9.07 (8.94, 9.19) |
| 55 | 7.40 (7.24, 7.57) | 8.36 (8.23, 8.48) | 9.10 (8.99, 9.21) |
| 60 | 7.46 (7.30, 7.63) | 8.41 (8.30, 8.52) | 9.14 (9.03, 9.25) |
| 65 | 7.53 (7.34, 7.72) | 8.47 (8.35, 8.58) | 9.18 (9.06, 9.30) |
| 70 | 7.59 (7.37, 7.81) | 8.52 (8.39, 8.66) | 9.22 (9.08, 9.35) |
| 75 | 7.65 (7.39, 7.91) | 8.58 (8.42, 8.74) | 9.26 (9.10, 9.41) |
| 80 | 7.71 (7.40, 8.02) | 8.64 (8.45, 8.82) | 9.30 (9.12, 9.48) |
| 85 | 7.77 (7.41, 8.13) | 8.69 (8.47, 8.91) | 9.33 (9.13, 9.54) |
| 90 | 7.84 (7.43, 8.25) | 8.75 (8.49, 9.01) | 9.37 (9.14, 9.61) |

**Supplementary Table 7**: Marginal effects on daily usage duration estimated with multivariable ordinal logistic regression. Four models were fitted, each using the respective total/subscale score as the first predictor. The odds ratios with their 95% confidence intervals are shown above, the *p*-values are shown below. Significant effects are shown in bold. The reference predictor levels were BTE for audio processor, bilateral CI for hearing configuration, female for gender, and mean age for age. Age estimates are given per 10-year increase beyond the mean age.

|  |  | **Total** | **Social life** | **Usability** | **Comfort** |
| --- | --- | --- | --- | --- | --- |
| **Score** |  | **0.69 (0.61, 0.79)** | **0.76 (0.69, 0.83)** | **0.83 (0.73, 0.94)** | **0.79 (0.72, 0.88)** |
|  |  | ***<.001*** | ***<.001*** | ***.003*** | ***<.001*** |
| **Audio processor type** | OTE | 1.26 (0.89, 1.77) | 1.18 (0.83, 1.67) | 1.30 (0.92, 1.83) | 1.30 (0.92, 1.84) |
|  |  | .193 | .354 | .137 | .131 |
| **Hearing configuration** | Unilateral CI | 1.36 (0.81, 2.27) | 1.32 (0.79, 2.20) | 1.31 (0.79, 2.18) | 1.34 (0.81, 2.24) |
|  |  | .241 | .292 | .297 | .256 |
|  | Bimodal | 1.49 (0.98, 2.26) | 1.41 (0.93, 2.15) | **1.57 (1.04, 2.37)** | **1.53 (1.01, 2.31)** |
|  |  | .062 | .107 | ***.032*** | ***.046*** |
|  | SSD | **2.78 (1.81, 4.27)** | **2.68 (1.74, 4.11)** | **2.91 (1.90, 4.45)** | **2.67 (1.75, 4.10)** |
|  |  | ***<.001*** | ***<.001*** | ***<.001*** | ***<.001*** |
| **Gender** | Male | 0.85 (0.61, 1.18) | 0.83 (0.60, 1.15) | 0.89 (0.65, 1.23) | 0.92 (0.67, 1.28) |
|  |  | .33 | .262 | .486 | .628 |
| **Age** | Per 10-year increase | 0.96 (0.87, 1.06) | 0.93 (0.84, 1.03) | 0.96 (0.87, 1.06) | 0.98 (0.89, 1.08) |
|  |  | .443 | .166 | .431 | .674 |

**Supplementary Table 8**: Effects on daily usage duration estimated with univariable ordinal logistic regression. Significant effects are shown in bold. The reference predictor levels were BTE for audio processor, bilateral CI for hearing configuration, female for gender, and mean age for age. Per decade indicates an additional decade of age beyond the mean age.

|  |  | **OR (95% CI)** | ***p*-value** |
| --- | --- | --- | --- |
| **Total score** |  | 0.70 (0.62, 0.79) | ***<.001*** |
| **Social life score** |  | 0.76 (0.70, 0.83) | ***<.001*** |
| **Usability score** |  | 0.87 (0.77, 0.98) | ***.021*** |
| **Comfort score** |  | 0.79 (0.72, 0.87) | ***<.001*** |
| **Audio processor type** | OTE | 1.47 (1.06, 2.04) | ***.022*** |
| **Hearing configuration** | Unilateral CI | 1.26 (0.76, 2.08) | .366 |
|  | Bimodal | 1.54 (1.03, 2.30) | ***.034*** |
|  | SSD | 2.92 (1.93, 4.43) | ***<.001*** |
| **Gender** | Male | 0.89 (0.66, 1.21) | .460 |
| **Age** | Per decade | 0.95 (0.86, 1.04) | .230 |
